# Preliminary cost analysis of prosthodontic rehabilitation in partial and complete edentulism: evidence from a private clinical setting in Greece

**DOI:** 10.3389/fpubh.2026.1779013

**Published:** 2026-02-04

**Authors:** Panagiota Chatzidou, Vasilios Stefos, Meira Chint, John Fanourgiakis

**Affiliations:** 1 Private Dental Practitioner, Thessaloniki, Greece; 2 Healthcare Management Program, School of Social Sciences, Hellenic Open University, Patras, Greece; 3 Department of Management Science and Technology, Hellenic Mediterranean University, Crete, Greece

**Keywords:** cost analysis, dental implants, dental restoration, fixed prosthodontics, Greece, health policy, removable prosthodontics, restoration cost

## Abstract

**Background:**

Oral health is fundamental to overall well-being; however, dental services in Greece are predominantly provided by the private sector, with limited public coverage, resulting in substantial out-of-pocket expenses and unmet treatment needs. This study presents the first comprehensive cost analysis of prosthodontic rehabilitation for partial and complete edentulism in Greece from the patient’s perspective.

**Methods:**

A retrospective micro-costing methodology was used for 76 patients treated at a private clinic between 2021 and 2022. Costs were categorised by treatment modality, including fixed prostheses (e.g., implants, conventional bridges, Maryland bridges, and combined techniques) and removable options (e.g., complete dentures, partial dentures, thermoplastic prostheses, and implant-supported overdentures).

**Results:**

The average cost per tooth varied widely: implants (€1,329.57), combined fixed restorations (€898.41), conventional bridges (€430.49), Maryland bridges (€356.25), partial dentures (€207.00), implant-supported overdentures (€202.21), thermoplastic prostheses (€79.38), and complete dentures (€77.92). Treatment duration ranged from approximately 0.4 months for Maryland bridges to over 6 months for implant procedures.

**Conclusion:**

These findings reveal notable cost disparities across treatment modalities and emphasise the financial burden associated with implant-based therapies. The results provide essential evidence for policymakers and healthcare professionals to guide reimbursement initiatives and improve access to prosthodontic care in Greece, where private expenditure is predominant.

## 1 Introduction

The provision of dental services, along with broader access to healthcare, is a crucial indicator of social equity and the efficiency of a healthcare system. In a European comparative context, Greece has historically faced considerable challenges, including inadequate dental care coverage, financial strain on households, and social disparities (1–3).

According to 2018 data, Greece ranks among the European Union (EU) countries with the highest unmet dental needs, at 14.0%. This figure is nearly double the EU average. In contrast, unmet medical care (8.0%) and pharmaceutical needs (7.0%) remain comparatively lower, indicating that dental care constitutes the most significant coverage gap (4). Social inequalities are most evident in dental care, where 23.0% of individuals in lower-income groups lack necessary treatment, compared with only 7.0% in higher-income groups (1, 2).

Regarding public expenditure, the state’s contribution to healthcare costs declined significantly after the 2008 financial crisis. Total healthcare expenditure as a percentage of Gross Domestic Product (GDP) fell from 9.5% in 2009 to 8.2% in 2015 and 8.4% in 2017. The crisis had a profoundly negative effect on public health financing, particularly affecting vulnerable populations (4). Public coverage of dental services in Greece (through EOPYY and other structures) is limited compared with Central and Northern European countries, where the public sector covers a broader range of dental services (2, 3).

Unmet dental needs in Greece have tripled between 2012 and 2022. Greece ranked second in the EU for unmet health needs in 2022. In countries such as Germany, the Netherlands, and Denmark, where government involvement in healthcare is substantial, the prevalence of unmet needs is considerably lower. The financial crisis, coupled with the restructuring of the Greek public health system, has exacerbated deficiencies in dental care (2, 4). Persistent underfunding and structural deficiencies in the Greek public health system are likely to exacerbate unmet needs and intensify social inequalities unless decisive measures are taken (2, 4).

Given the predominance of private provision and out-of-pocket financing, financial barriers translate directly into foregone preventive and restorative care, particularly among economically disadvantaged households (5). Cost remains the primary driver of unmet dental care needs, outweighing considerations of geographic distance or waiting times. Economically disadvantaged households bear a disproportionate burden, which not only reduces preventive care services, such as dental cleanings and basic restorations, but also increases the likelihood of more severe health complications and secondary costs within the healthcare system (2, 6, 7).

A comprehensive literature review identified 13 studies across 11 countries that estimate the costs of dental restorations (Appendix Table A). No studies focusing exclusively on Greek costs were available. The only available Greek-related evidence derives from a preliminary cost–utility analysis conducted in dental practices in the greater Athens area, reported in an unpublished master’s thesis entitled “Cost–utility study in patients who have undergone dental implant placement” (8). International analyses of costs demonstrate considerable variability, contingent on treatment choices and the specified time horizon. For replacing a single tooth, implants are generally more cost-effective than conventional fixed bridges. Studies from Switzerland indicated that the initial cost of a crown placed on an implant was lower (3,218 SFr and 4,498 CHF, respectively) than that of a traditional bridge (3,939.4 SFr and 5,082 CHF), primarily owing to reduced laboratory expenses (9, 10).

Similarly, in South Korea, the initial direct costs of bridges (1,292,960 KRW) and implants (1,339,170 KRW) were comparable. From a cost-effectiveness perspective, implants become more economical around the tenth year of use. This finding aligns with the Greek study, which calculated a ten-year cost of €1,826.00 for an implant versus €1,897.00 for a bridge (8). In the Czech Republic, although the initial costs were nearly identical (40,286 CZK for implants versus 40,161 CZK for bridges), a thirty-year analysis indicated that implants were the more economical option (11). In Japan, insurance coverage significantly affected costs: bridges ranged from €420.00 (insured) to €2,618.00 (private), compared with €2,744.00 for implants (12). In the United States, indicative costs for a single implant are approximately $3,500.00, with a single crown costing approximately $630.00–$640.00 (13), implying a bridge cost of approximately $1,905.00.

For full-arch rehabilitation, traditional complete dentures remain the most economical option initially, with costs approximating €1,800.00 in Italy and €1,060.00 in the Netherlands. Nevertheless, implant-retained overdentures supported by two implants have proven the most cost-effective approach, offering a substantial improvement in quality of life at intermediate costs (€3,441.00 in the Netherlands; €4,473.00 in Italy; €4,519.00 in Sweden) (14–16). Fixed full-arch implant-supported restorations are the most expensive treatment modality, with four-implant bridges priced between €9,300.00 and €10,000.00, and six-implant solutions exceeding €11,400.00 (15, 16).

Laboratory costs highlight the economic implications of digital workflows (Appendix Table B). Research in Switzerland and Italy shows that digital workflows reduce laboratory expenditure: a single implant crown in Italy costs €277.00 when produced digitally, compared with €392.00 using conventional techniques. Furthermore, a three-unit implant bridge in Switzerland costs between 566.00 and 711.00 CHF when produced digitally, compared with 812.00 CHF through hybrid workflows (17). Correspondingly, the costs of full dentures in Italy have decreased from $270.00 using traditional methods to $184.00 when fabricated through digital milling, and further to $67.00 via 3D printing (18).

Oral health is fundamental to overall health, well-being, and psychological resilience. In Greece, dental services are predominantly provided by the private sector, with limited coverage under public insurance schemes, resulting in high out-of-pocket costs for patients and unmet dental care needs, particularly for costly interventions such as prosthodontic rehabilitation (2, 4, 6, 7). However, comprehensive, context-specific data remain scarce, revealing a substantial knowledge gap regarding the financial burden of prosthodontic care in Greece. To the best of our knowledge, no published Greek economic evaluation has examined the full spectrum of prosthodontic rehabilitation for partial or complete edentulism. This gap underscores the pressing need for robust, contemporary cost data to support evidence-based decision-making, particularly within a healthcare system characterised by predominantly private dental service provision and high out-of-pocket expenditure (2, 4).

The present study aimed to estimate the mean total cost of various prosthodontic rehabilitation options for patients with missing teeth, encompassing both fixed and removable prosthetic solutions. Fixed restorations included dental implants, conventional fixed bridges, Maryland-type bridges, and combined approaches integrating conventional prostheses with implants. Removable prostheses comprised conventional complete dentures, partial dentures, telescopic dentures, implant-retained overdentures, and prostheses fabricated from thermoplastic materials. The analysis further considered factors influencing treatment costs, including the number of teeth involved in the final restoration, the aetiology of tooth loss, patient sex and age, material and laboratory costs, pre-prosthetic procedures, total treatment duration, and opportunity costs related to time spent at the dental clinic. The overarching aim was to provide a reference framework for estimating dental care costs in Greece, relevant for both patients and healthcare providers.

## 2 Materials and methods

A comparative cost analysis of various treatment modalities was conducted using a bottom-up micro-costing approach from the perspective of private patient expenditure. Primary data were obtained from a private dental clinic, including invoices, medical records, and appointment management documents. All data was anonymised by removing personal and identifiable information for both patients and clinicians in accordance with prevailing data protection standards, thereby ensuring confidentiality and compliance with ethical standards.

The study population initially comprised 89 patients; after applying the exclusion criteria, 13 were excluded, leaving a final sample of 76. Inclusion criteria included completion of tooth-restorative treatment between 2021 and 2022, whereas exclusion criteria included incomplete treatment, lack of consent for data processing, and treatments not involving tooth loss. Demographic and clinical data recorded for each patient included age, sex, cause of tooth loss, type of prosthetic restoration, number of missing teeth, number of treated teeth, treatment duration, and economic variables related to each treatment component. The cost components evaluated encompassed pre-prosthetic procedures, provisional restorations, materials, laboratory work, surgical procedures, prosthetic procedures, and total patient costs. Opportunity costs were also calculated based on the total time patients spent attending appointments.

Material costs for each dental procedure were based on purchase invoices collected over the study period, with treatment costs allocated per patient. Implant-related components, including abutments and healing caps, were priced using supplier invoices. Laboratory costs were calculated from invoices received from collaborating dental laboratories. Pre-prosthetic costs were determined by summing invoices for preparatory procedures, while surgical procedure costs included both materials, such as implants, surgical drills, membranes, and grafts, and the total charges billed to patients. Prosthetic procedure costs were obtained from the final patient invoices. Total patient costs were defined as the sum of pre-prosthetic, surgical, and prosthetic costs, covering all applicable expenses according to the clinic’s pricing policy.

The treatment duration was defined as the period between the initial assessment and the final intervention, with days converted to months for comparative analysis (1 month = 30 days). The total time patients spent in the clinic was calculated as the sum of individual appointment durations.

The data were consolidated into an Excel database, and statistical analyses were conducted using SPSS software (version 31.0, SPSS, Inc., IBM Corp., Armonk, NY, United States).

Descriptive statistics included mean, standard deviation (SD), median, and minimum–maximum values, while normality of the distribution was examined using the Kolmogorov–Smirnov and Shapiro–Wilk tests. Treatment duration and time spent in the clinic for each prosthetic modality were summarised using mean, median, standard deviation, and range. To evaluate the relationship between overall patient cost and the type of prosthetic restoration, a Generalised Linear Model (Gamma GLM) with a logarithmic link function was utilised, with statistical significance established at *p* < 0.05. By providing this comprehensive cost analysis from a private clinical setting in Greece, the study addresses a notable gap in national evidence and offers vital data to inform healthcare policy, reimbursement strategies, and patient financial planning within the scope of prosthodontic care.

The Institutional Review Board granted ethical approval under protocol number 162891/13-11-2024, and the study complied with the principles outlined in the Declaration of Helsinki (2013 revision).

## 3 Results

A total of 76 patients met the study’s inclusion criteria, comprising a diverse group undergoing prosthodontic rehabilitation for partial or complete edentulism at a private Greek dental clinic. Of these, 37 were male (48.7%) and 39 were female (51.3%), resulting in a balanced gender distribution (Table 1).

**Table 1:** Distribution of patients by sex, in the study population (*N* = 76).

Sex	Frequency (N)	Percentage (%)
Male	37	48.7
Female	39	51.3
Total	76	100.0

The mean age of patients was 59.17 years (SD = 12.55), with a range from 23.0 to 86.0 years. This wide age range underscores the clinical importance of prosthodontic rehabilitation for both younger adults and older populations.

The dental status of patients varied considerably. On average, patients had 7.08 missing teeth (range: 1–32) and received restorations for 6.71 teeth (range: 1–28), indicating that the number of restored teeth did not always match the number of missing teeth. This difference reflects clinical decision-making influenced by treatment planning, patient preference, oral health condition, and the type of prosthetic intervention chosen. Table 2 provides descriptive statistics for age, missing teeth, and teeth restored.

**Table 2:** Age, missing teeth, and restored teeth in the study population (*N* = 76).

Variable	Mean	Min	Max	SD	Range
Patient age (years)	59.17	23	86	12.55	63
Missing teeth	7.08	1	32	8.76	31
Teeth restored	6.71	1	28	6.44	27

Appendix Table C further clarifies the relationship between the number of teeth missing and the number of teeth restored, emphasising the inherent variability in prosthodontic treatment planning.

The primary causes of tooth loss within the cohort were categorised into six groups. Periodontal disease was identified as the most prevalent factor, affecting 21 patients (27.6%), followed by dental fractures in 16 cases (21.1%) and complications arising from endodontic therapy in 13 cases (17.1%). Dental caries was the causative factor in 6 patients (7.9%), whereas congenital absence was infrequent, observed in only one patient (1.3%). A significant proportion of patients, equaling 18 individuals (23.7%), exhibited multiple contributory factors to tooth loss, thereby exemplifying the multifactorial etiology commonly encountered in clinical practice. Furthermore, 1 patient (1.3%) underwent rehabilitation to replace a previously unsatisfactory prosthesis (Table 3).

**Table 3:** Causes of tooth loss in the study population (*N* = 76).

Cause of tooth loss	Frequency (N)	Percentage (%)
Periodontal disease	21	27.6
Combination of factors	18	23.7
Fracture	16	21.1
Complications of endodontic treatment	13	17.1
Caries	6	7.9
Replacement of previous prosthesis	1	1.3
Congenital absence	1	1.3

These results underscore the complex interplay of biological, traumatic, and iatrogenic factors contributing to tooth loss and highlight the importance of individualised treatment planning.

The distribution of prosthetic interventions reflects both patients’ clinical needs and common practices in private dental care. Among the 76 patients, 57 (75%) received fixed prosthetic restorations, including 27 rehabilitated solely with dental implants (35.53%), 18 with conventional dental bridges (23.68%), 2 with Maryland-type bridges (2.63%), and 10 receiving combined fixed restorations incorporating implants (13.16%) (Appendix Table D).

The remaining 19 patients (25.00%) received removable prosthetic treatments, including 9 complete dentures (11.85%), 6 partial dentures (7.89%), 2 thermoplastic prostheses (2.63%), and 2 implant-supported overdentures (2.63%) (Table 4).

**Table 4:** Removable prosthetic treatment modalities in the study population (*N* = 76).

Number of teeth	Implant-supported overdentures	Partial dentures	Thermoplastic dentures	Complete dentures	Total cases
6	0	1	0	0	1
8	0	0	1	1	2
10	0	3	1	0	4
12	0	1	0	0	1
14	2	0	0	6	8
28	0	1	0	2	3
Total	2	6	2	9	19
Percentage of total (*N* = 76)	2.63%	7.89%	2.63%	11.85%	25.00%

Notably, although removable prostheses were less commonly used, they were often employed to restore a greater number of teeth per treatment, highlighting their suitability for patients with extensive edentulism and their versatility in full-arch rehabilitation.

Treatment duration, encompassing both total completion time and chairside time, was profoundly affected by the type and complexity of the prosthetic restoration. Implant-based restorations had a mean total duration of 6.30 months (SD = 5.11), with a mean chairside time of 5.26 h (SD = 0.45). Implant-supported overdentures required approximately 5.00 months for completion, involving 6.50 h of chairside work, reflecting the surgical and prosthetic procedures entailed. Thermoplastic prostheses, in contrast, achieved completion within 1.50 months and required only 2.00 h of chairside engagement, demonstrating their efficiency for less complex cases. Partial dentures required 2.43 months and 6.83 h, while complete dentures were finalised in 1.33 months and 4.56 h of chairside time. Combined fixed restorations with implants required 5.90 months and 6.90 h, whereas conventional dental bridges were completed in 1.62 months and 6.17 h in the dental chair. Maryland bridges required the shortest overall duration (0.40 months) and minimal chairside involvement (2.50 h), reflecting their simpler design and reduced clinical steps (Table 5). Standard error refers to the SE of the mean.

**Table 5:** Treatment duration (complete time) and (chairside time) in the study population, (in months), (*N* = 76).

Restoration type	Completion time (months)	Minimum	Maximum	Median	Standard error		Chairside time (hours)	Minimum	Maximum	Median	Standard Error
Implants	6.30	3.00	12.00	6.00	0.51		5.26	2	11	5.00	0.45
Implant-supported overdentures	5.00	5.00	5.00	5.00	0.00		6.50	6	7	6.50	0.50
Thermoplastic prostheses	1.50	1.00	2.00	1.50	0.50		2.00	2	2	2.00	0.00
Partial dentures	2.43	2.00	3.00	2.40	0.20		6.83	5	8	7.00	0.47
Complete dentures	1.33	0.00	3.00	1.00	0.28		4.56	2	6	4.00	0.44
Combined fixed restorations	5.90	4.00	9.00	6.00	0.43		6.90	3	12	7.00	0.99
Conventional bridges	1.62	0.00	3.00	1.80	0.18		6.17	2	14	5.00	0.75
Maryland bridges	0.40	0.00	1.00	0.40	0.10		2.50	2	3	2.50	0.50

These findings highlight considerable variation in treatment timelines and resource utilisation across diverse prosthetic modalities.

Pre-prosthetic procedures, including diagnostic imaging, optimisation of oral health, surgical preparation, and fabrication of temporary restorations, showed significant variability in both frequency and cost. Within the implant category, the mean cost of pre-prosthetic procedures per restored tooth was €22.22 (SD = 72.50), with a maximum expenditure of €350.00. Implant-supported overdentures had a higher mean cost of €225.00 (SD = 318.20), while partial dentures averaged €236.67 (SD = 184.03), reflecting the typical preparatory work required to ensure stable abutment teeth. Complete dentures incurred a mean pre-prosthetic cost of €111.11 (SD = 96.10). In contrast, combined fixed restorations with implants had a mean cost of €360.00 (SD = 404.01), indicative of considerable variability attributable to patient-specific factors and procedural complexity. Conventional dental bridges had a mean cost of €333.33 (SD = 435.81), whereas Maryland bridges and thermoplastic prostheses had no pre-prosthetic costs. The broad range of values in specific categories underscores the varying financial implications of preparatory procedures, especially in more complex restorative cases (Table 6).

**Table 6:** Mean cost of pre-prosthetic procedures (€), in the study population (*N* = 76).

Prosthetic category	Mean (€)	Minimum (€)	Maximum (€)	SD	Standard error
Implants	22.22	0.00	350.00	72.50	13.95
Implant-supported overdentures	225.00	0.00	450.00	318.20	225.00
Partial dentures	236.67	0.00	520.00	184.03	75.13
Complete dentures	111.11	0.00	250.00	96.11	32.03
Combined implants and fixed restorations	360.00	0.00	1,250.00	404.01	127.75
Conventional dental bridges	333.33	0.00	1,800.00	435.81	102.72
Thermoplastic prostheses	0.00	0.00	0.00	0.00	0.00
Maryland bridges	0.00	0.00	0.00	0.00	0.00

Material costs, including impression materials, temporary and permanent cements, provisional restoration components, and prosthetic parts for implant-supported restorations, showed comparable variability across modalities. Implant restorations had the highest mean material cost per restored tooth (€108.65, SD = 58.13), followed by combined implants with other fixed restorations (€78.81, SD = 55.23). Maryland bridges and implant-supported overdentures averaged €18.75 and €16.79, respectively. Conventional bridges cost €11.68 per tooth, partial dentures €5.54, thermoplastic removable prostheses €4.38, and complete dentures €3.31. These findings emphasise that material costs are predominantly influenced by the type and complexity of the restoration, with implant-based procedures requiring significantly greater resources than conventional removable solutions (Table 7).

**Table 7:** Material costs per restored tooth (in €), in the study population (*N* = 76).

Type of restoration	Mean material cost per tooth (in €)	Range (in €) (Min–Max)	SD
Implants	108.65	41.25–245.00	58.13
Combined implants and other fixed restorations	78.81	25.00–198.80	55.23
Maryland bridges	18.75	7.50–30.00	15.91
Implant-supported overdentures	16.79	15.71–17.86	1.52
Conventional dental bridges	11.68	2.35–40.00	7.80
Partial dentures	5.54	4.17–8.33	1.49
Removable thermoplastic prostheses	4.38	3.75–5.00	0.88
Complete dentures	3.31	1.43–6.25	1.30

Laboratory collaboration is a vital element of prosthetic treatment, influencing both financial expenditure and quality of care. The mean laboratory cost per restored tooth was highest for combined implants and other fixed restorations (€118.26, SD = 77.44) and for implants (€106.08, SD = 43.97). In contrast, complete dentures incurred the lowest mean laboratory cost (€27.14, SD = 9.54). Maryland bridges had a mean laboratory cost of €57.50 (SD = 60.10), while implant-supported overdentures, partial/telescopic dentures, and thermoplastic removable prostheses showed intermediate costs (€39.29, €60.84, and €30.50, respectively). Conventional bridges averaged €85.87 (SD = 45.69), reflecting considerable variability across laboratories and the complexity of the design (Table 8).

**Table 8:** Laboratory collaboration cost per restored tooth (in €) in the study population (*N* = 76).

Category	Mean (€)	Minimum (€)	Maximum (€)	SD
Conventional bridges	85.87	21.67	245.00	45.69
Implants	106.08	30.00	235.00	43.97
Implants and other fixed restorations	118.26	45.00	266.25	77.44
Maryland bridges	57.50	15.00	100.00	60.10
Complete Dentures	27.14	8.57	45.00	9.54
Implant-supported overdentures	39.29	35.70	42.85	5.05
Partial/telescopic dentures	60.84	35.70	110.00	27.60
Removable thermoplastic prostheses	30.50	15.00	46.00	21.92

The cost of prosthetic procedures, including laboratory and material expenses but excluding pre-prosthetic and surgical costs, varied considerably across treatment modalities. Implants incurred the highest mean cost per restored tooth (€676.24, SD = 275.77), followed by combined implants with other fixed restorations (€538.13, SD = 254.58). Conventional bridges and Maryland bridges had comparable costs at €368.22 (SD = 211.13) and €356.25 (SD = 344.71), respectively. Partial dentures (€185.57, SD = 94.33) and implant-supported overdentures (€151.42, SD = 72.73) were more cost-effective, whereas thermoplastic prostheses (€79.38, SD = 15.03) and complete dentures (€71.57, SD = 26.02) were the least expensive options. The higher standard deviations observed in complex restorations such as implants reflect the heterogeneity of clinical requirements, whereas simpler prostheses exhibited narrower cost distributions (Table 9).

**Table 9:** Prosthetic cost per restored tooth (in €), in the study population (N = 76).

Category	Mean (€)	Minimum (€)	Maximum (€)	SD
Conventional bridges	368.22	60.00	1,050.00	211.13
Implants	676.24	190.00	1,390.00	275.77
Implants and other fixed restorations	538.13	285.00	955.00	254.58
Maryland bridges	356.25	112.50	600.00	344.71
Complete dentures	71.57	32.14	114.29	26.02
Partial dentures	185.57	62.50	283.33	94.33
Removable thermoplastic prostheses	79.38	68.75	90.00	15.03
Implant-supported overdentures	151.42	100.00	202.85	72.73

Surgical material costs, including implants, grafts, and sutures, were primarily relevant to implant-based restorations. The mean surgical material expenditure for single-tooth implants was €309.10 (SD = 116.50), for combined fixed restorations with implants €152.63 (SD = 60.16), and for implant-supported overdentures €51.78 (SD = 0.50). Expenses related to surgical materials for other prosthetic modalities, including conventional bridges, Maryland bridges, complete dentures, partial dentures, and thermoplastic removable prostheses, were negligible because no surgical procedures were performed (refer to Table 10).

**Table 10:** Surgical material cost per restored tooth (in €), in the study population (*N* = 76).

Category	Mean (€)	Minimum (€)	Maximum (€)	SD
Conventional bridges	0.00	0.00	0.00	0.00
Implants	309.10	101.25	520.00	116.50
Implants and other fixed restorations	152.63	42.30	243.00	60.16
Implant-supported overdentures	51.78	51.42	52.14	0.50
Maryland bridges	0.00	0.00	0.00	0.00
Complete dentures	0.00	0.00	0.00	0.00
Partial/telescopic dentures	0.00	0.00	0.00	0.00
Removable thermoplastic prostheses	0.00	0.00	0.00	0.00

Patient-borne surgical costs, including both material and procedural charges, reflected these differences. Implants had an average surgical cost of €647.76 (SD = 210.14), with a range of €200.00–1,100.00. Combined fixed restorations cost €297.36 (SD = 107.56), and implant-supported overdentures cost €135.71 (SD = 30.30). Other prosthetic modalities incurred no surgical costs (Table 11).

**Table 11:** Surgical cost per restored tooth (in €).

Category	Mean (€)	Minimum (€)	Maximum (€)	SD
Conventional bridges	0.00	0.00	0.00	0.00
Implants	647.76	200.00	1,100.00	210.14
Implants and other fixed restorations	297.36	120.00	425.00	107.56
Implant-supported overdentures	135.71	114.28	157.14	30.30
Maryland bridges	0.00	0.00	0.00	0.00
Complete dentures	0.00	0.00	0.00	0.00
Partial dentures	0.00	0.00	0.00	0.00
Removable thermoplastic prostheses	0.00	0.00	0.00	0.00

These data highlight the substantial financial impact of surgical intervention on the total costs of implant-based treatments.

The mean total cost per restored tooth, reflecting the sum of pre-prosthetic, material, laboratory, prosthetic, and surgical costs, varied widely across treatment modalities. Single-tooth implants incurred the highest costs at €1,329.57 (SD = 426.52, range €390.00–2,290.00), followed by combined implants with other fixed restorations (€898.41, SD = 373.00, range €405.00–1,447.00), conventional bridges (€430.49, SD = 241.41, range €60.00–1,200.00), and Maryland bridges (€356.25, SD = 344.71, range €112.50–600.00). Removable prostheses were significantly more cost-effective, with partial dentures (€207.00, SD = 104.46, range €79.17–341.67), implant-supported overdentures (€202.21, SD = 65.15, range €257.14–349.28), thermoplastic prostheses (€79.38, SD = 15.03, range €68.75–90.00), and complete dentures (€77.92, SD = 28.82, range €32.14–132.14) representing the most economical options (see Table 12).

**Table 12:** Mean total cost per restored tooth (in €).

Category	Mean (€)	Minimum (€)	Maximum (€)	SD
Implants	1,329.57	390.00	2,290.00	426.52
Implants and other fixed restorations	898.41	405.00	1,447.00	373.00
Conventional bridges	430.49	60.00	1,200.00	241.41
Maryland bridges	356.25	112.50	600.00	344.71
Partial dentures	206.70	79.17	341.67	104.46
Implant-supported overdentures	202.21	257.14	349.28	65.15
Removable thermoplastic prostheses	79.38	68.75	90.00	15.07
Complete dentures	77.92	32.14	132.14	28.82

To assess the statistical significance of cost disparities across restoration types, a Generalised Linear Model (Gamma GLM) with a log link function was used, with complete dentures as the reference category because they are the least costly. This approach was deemed appropriate given the non-normal, positively skewed distribution of cost data, as confirmed by Kolmogorov–Smirnov and Shapiro–Wilk tests. The results indicated significant differences (*p* < 0.01) in mean cost per restored tooth across all prosthetic categories, except for thermoplastic removable prostheses. Single-tooth implants were associated with a 17.06-fold increase in cost relative to complete dentures (+1,606.4%), while combined fixed restorations with implants showed an 11.53-fold increase (+1,053.0%). Conventional bridges exhibited a 5.53-fold increase (+454.5%), Maryland bridges a 4.57-fold increase (+357.2%), implant-supported overdentures a 3.89-fold increase (+289.2%), and partial dentures a 2.66-fold increase (+165.7%). Thermoplastic prostheses showed no statistically significant difference (+1.9%) (Table 13).

**Table 13:** Percentage increase per restored tooth in cost relative to reference restoration.

Restoration type	B	Exp(B)	Cost multiplier	% Increase
Complete dentures (Reference)	0.00	1.000	1.0×	0.0%
Implants	2.837	17.064	17.1×	+1,606.4%
Implants and other fixed restorations	2.445	11.530	11.5×	+1,053.0%
Conventional bridges	1.709	5.525	5.5×	+452.5%
Maryland bridges	1.520	4.572	4.6×	+357.2%
Implant-supported overdentures	1.359	3.892	3.9×	+289.2%
Partial dentures	0.977	2.657	2.7×	+165.7%
Thermoplastic removable prostheses	0.019	1.019	1.0×	+1.9%

These findings underscore the considerable influence of restoration type on patient expenditure, reflecting both clinical complexity and material requirements.

In summary, the results reveal significant heterogeneity in the clinical and financial aspects of prosthodontic rehabilitation. Fixed implant-based restorations are associated with the highest procedural complexity, chairside and total completion times, and costs. Removable prostheses, including complete dentures, are the least complex and least costly, while thermoplastic options offer a lower-cost alternative for patients with extensive tooth loss. The variability in costs within each category, particularly for implants and combined fixed restorations, highlights the individualised nature of prosthodontic treatment planning and the impact of patient-specific factors such as the number of teeth restored, pre-prosthetic needs, and the choice of materials and laboratory techniques.

These findings, for the first time in Greece, provide a detailed, stratified estimate of the economic burden of prosthodontic rehabilitation, offering vital information for both clinicians and policymakers. They emphasise the importance of incorporating cost considerations into treatment planning, patient counselling, and broader assessments of oral healthcare accessibility, particularly in contexts where private expenditure predominates. The data also indicate that while implant-based therapies provide functional and aesthetic benefits, cost remains a significant barrier for many patients, underlining the need for customised treatment strategies that balance clinical outcomes with economic feasibility.

## 4 Discussion

The findings of this study offer a comprehensive economic and clinical evaluation of prosthodontic rehabilitation within a private dental practice in Greece, reflecting both the financial and temporal burdens borne by patients. Socioeconomic factors are critical in determining access to dental care, as emphasised by Bourazani and Konstantinidis and the Organisation for Economic Co-operation and Development (OECD) (2019, 2022) (2, 4, 19), who highlight that a patient’s financial capacity significantly influences their treatment options. In this analysis, treatment costs were primarily influenced by the complexity of prosthetic procedures, reflecting how procedural complexity translates into financial barriers within privately financed dental systems (2, 20).

A comprehensive cost analysis found that single-tooth implants cost a mean of €1,329.57, marginally higher than traditional bridges at €1,291.46, which generally replace three teeth. These results align with a previous Greek study (8), indicating minimal long-term cost differences between implants (€1,826.00) and bridges (€1,897.00). Conversely, Swiss studies reported lower costs for single-tooth implants (€2,100.00–€2,900.00) than for conventional bridges (€2,600.00–€3,300.00), highlighting country-specific differences in pricing, healthcare infrastructure, and material costs (9, 10). Such variations underscore the importance of context-specific economic evaluations in prosthodontics (21, 22).

For complete edentulism, conventional dentures were the most cost-effective, averaging €1,247.52 per 16-tooth arch, consistent with international data (14, 23). This reinforces conventional dentures as the primary affordable option for complete edentulism in settings without public coverage. Implant-supported overdentures were more expensive (€3,232.00 per arch) but remained within the reported range of €4,500–€4,600 (14, 16). Complex fixed restorations using multiple implants (All-on-4/6) incurred the highest mean cost (€7,977.42), aligning with global reports (€9,300–€11,400) (15, 24). These results confirm a global cost gradient in prosthodontics, driven mainly by procedural complexity, implant quantity, and materials (12, 25, 26).

Moreover, thermoplastic partial dentures were less expensive (€725.00) than conventional metal-acrylic alternatives (€1,708.00), contrary to findings in other contexts (23). This discrepancy likely reflects differences in case complexity; conventional dentures often required extensive pre-prosthetic procedures, including endodontic and fixed restorations (mean €520.00), whereas thermoplastic solutions were selected for simpler rehabilitations. These observations underscore the influence of clinical context on cost structures (11, 27, 28).

The study presents a predictive cost model using a Gamma Generalised Linear Model (GLM) with a log link, quantifying expenditure relative to complete dentures. This steep cost gradient underscores how material intensity and surgical involvement amplify patient expenditure, complementing prior models that emphasised cost multipliers in prosthodontics (8–10). Longer chairside and completion times further compound the opportunity costs associated with complex fixed restorations, highlighting the broader patient burden, consistent with findings on time efficiency in both digital and conventional workflows (17, 18, 29, 30).

Clinically, the dominance of preventable causes underscores the long-term economic value of preventive oral health strategies, highlighting the importance of preventive measures (6, 7, 31). These findings further reinforce the case for public health initiatives to reduce prosthetic demand and alleviate long-term economic burdens (3, 5). Additionally, socioeconomic factors significantly influence willingness to pay and access to advanced prosthodontic treatments (13, 32–34), raising concerns about equity in private dental practice settings (35–37).

Comparison with the international literature confirms that implant-based treatments are consistently the costliest, reflecting global patterns in material, labour, and procedural costs (14–16). In contrast, complete dentures remain the most cost-effective option (14–16). The reversal of cost patterns for partial dentures relative to thermoplastic alternatives underscores the importance of case selection, treatment complexity, and pre-prosthetic procedures (23, 27).

Limitations of the present study include reliance on a single-practice data source, which limits the generalisability of the findings, and small sample sizes for specific modalities, such as Maryland bridges, thermoplastic dentures, and implant-supported overdentures, which may affect the accuracy of the results. Furthermore, long-term maintenance, complication management, and prosthesis longevity were not addressed, despite their significance for comprehensive cost-effectiveness evaluations (12, 25, 26). Future research should involve multicentre, longitudinal studies that incorporate patient-reported outcomes and quality-of-life metrics to enhance economic analyses (13, 17, 32).

From a policy perspective, these findings provide a framework to support evidence-based decision-making. Clinicians may use cost hierarchies and predictive modelling to inform patients about anticipated costs and treatment planning. Meanwhile, policymakers could consider implementing subsidies or tiered insurance coverage to improve access to implant-based care (19, 38–40). Additionally, including opportunity costs further promotes patient-centred approaches that consider both financial and temporal burdens (17, 18).

According to the Organisation for Economic Co-operation and Development (OECD), in 2021, public financing (government and compulsory insurance) for dental care expenditure in Greece was negligible, whereas the average across OECD member countries was 32.99% (41).

According to the most recent Eurostat data (2024), 6.3% of individuals aged 16 years and over in the European Union who needed dental care did not receive it because of financial constraints, long waiting lists, or distance from dental care providers. Among EU countries, Greece records the highest proportion of unmet dental care needs (27.1%), followed by Latvia (16.5%) and Romania (16.2%). The lowest levels were observed in Malta (0.4%), Germany (0.9%), and Croatia (1.1%) (42).

The World Health Organization (WHO) (43, 44). recognises oral health as a fundamental component of overall health, quality of life, and social well-being. Oral diseases are among the most prevalent non-communicable diseases worldwide, affecting individuals across the life course and accounting for approximately 5.3% of global disability-adjusted life years (DALYs) (43, 44). Poor oral health and inadequate access to dental care are associated with pain, functional limitations, psychosocial consequences, reduced work productivity, and poorer educational outcomes, while oral diseases frequently coexist with other non-communicable diseases such as diabetes and cardiovascular disease (43, 44).

Tooth loss, whether partial or complete, represents one of the most disabling outcomes of oral disease. Partial and complete edentulism impair mastication, speech, aesthetics, and nutrition, leading to reduced quality of life and social participation. Oral rehabilitation should therefore be considered not merely a restorative intervention, but a critical public health measure aimed at restoring function, dignity, and social inclusion. Nevertheless, rehabilitative dental care is often costly and places a substantial financial burden on households.

In Greece, dental care has been disproportionately affected by the prolonged economic crisis and the COVID-19 pandemic. Between 2008 and 2021, public expenditure on dental care declined by 99.98% (45). As a result, public dental expenditure accounted for 0% of total dental spending in 2019, compared with an EU average of 31% (46). At the same time, private health expenditures decreased by nearly 72% (45). The public dental sector remains understaffed and under-resourced, contributing to widening inequalities and deteriorating oral health outcomes. Unmet dental care needs in Greece are significantly higher than the EU average (7.8% vs. 3.1% in 2021) (46) [whereas, respectively, 27,1% vs. 16,2% in 2024 (42)]. particularly among lower-income populations, reflecting a persistent social gradient in access to dental services (46). These findings highlight the urgent need to strengthen access to comprehensive rehabilitation for partial and complete edentulism.

To reduce unmet dental care needs, mitigate the risk of catastrophic health expenditures, improve oral health, and enhance the financial sustainability of the National Health System, and within the framework of implementing the objectives of the 2030 Agenda for Sustainable Development—which has been adopted by all United Nations Member States—it is proposed that the following policy options for dental care in Greece needs to be examined and adopted (47):

1. Investment in the prevention and early treatment of oral diseases, including diseases of the teeth.
2. Standardisation (coding), pricing, and reimbursement of dental materials and consumables based on their effectiveness or value, as documented within the framework of Health Technology Assessment (HTA).
3. Transparent pricing for medical and dental laboratory procedures and services.
4. Full or partial public reimbursement or subsidisation of dental care (or of a basic basket of services) for individuals, based on income criteria, to be determined following an actuarial study.
5. Electronic recording of dental procedures, services, and materials in each patient’s medical record, and integration of this information with IDIKA’s central electronic system to enhance transparency and enable real-time monitoring of expenditures.
6. Strengthening of the National Public Health System through the recruitment of dentists and dental technicians to reduce patient waiting times.
7. Development of teledentistry programmes for the early identification and management of oral health problems in populations facing access barriers, including individuals with mobility impairments, island residents and those in remote or hard-to-reach areas, and populations living in camps (e.g., migrants, Roma).
8. Development and operation of Mobile Oral Health Units aim to provide assessment, preventive care, and rehabilitation for individuals with partial or full edentulism, especially populations facing barriers like older adults, impoverished communities, refugees, Roma, and those with chronic illnesses, mental health issues, or substance dependence.

## 5 Conclusion

This study presents a robust economic and clinical evaluation of prosthodontic rehabilitation in Greek private practice. Key conclusions include:

1. *Cost hierarchy*: Total patient costs are predominantly influenced by the type and complexity of the prosthetic intervention, with implant-based restorations representing the most expensive option, while conventional removable or conservative fixed solutions are comparatively more affordable (8–10).
2. *Treatment duration and opportunity costs*: Implant-supported and surgically intensive restorations require substantially longer treatment periods (mean 5–6 months) than fixed bridges and removable prostheses (mean 1–2.5 months), reflecting increased procedural complexity and associated indirect costs (17, 18, 48).
3. *Predictive modelling*: The Gamma Generalised Linear Model (GLM) enables estimation of patient expenditure by restoration type, demonstrating approximately 17-fold higher costs for implant-based treatments, 5.5-fold increases for conventional bridges, and 2.7-fold increases for partial dentures.
4. *Clinical implications*: The primary causes of tooth loss highlighted in this study reinforce the critical role of preventive care in reducing long-term clinical, economic, and societal burdens (6, 31).
5. *Research and policy relevance*: The findings provide a reference framework for patient-borne costs in Greece, supporting evidence-based clinical decision-making, patient counselling, and the design of policy interventions aimed at improving access to advanced prosthodontic care (19, 38, 39).
6. *Health system and policy implications*: To address Greece’s unmet dental needs and financial barriers, improve oral health outcomes, and ensure system sustainability aligned with the 2030 Agenda, a comprehensive policy is needed. This should integrate prevention, value-based pricing, transparent reimbursement, income-based coverage, digital monitoring, workforce strengthening, and the expansion of teledentistry (42–47).

In conclusion, prosthodontic rehabilitation in Greece exhibits a clear cost gradient influenced by clinical complexity, material selection, and patient time investment. While implants deliver superior functional and aesthetic outcomes, their higher costs necessitate careful patient-centred planning. Future research should extend to larger, multicentre populations to validate these findings and support their generalisability across diverse healthcare settings (49).

## Data Availability

The raw data supporting the conclusions of this article will be made available by the authors, without undue reservation.

## Glossary

Periodontal Disease: A group of inflammatory diseases affecting the supporting tissues of the teeth, characterised by loss of periodontal ligament and alveolar bone, potentially leading to tooth mobility and tooth loss.
Combination of Factors: The presence of two or more aetiological or contributory conditions acting together to influence diagnosis, prognosis, or treatment planning (e.g., caries, periodontal disease, occlusal factors).
Fracture: A structural discontinuity or break in a tooth, restoration, or prosthesis, which may involve enamel, dentine, root structure, or prosthetic materials.
Complications of Endodontic Treatment: Adverse outcomes arising during or after root canal therapy, including but not limited to persistent infection, perforation, instrument separation, inadequate obturation, or post-treatment disease.
Caries: A biofilm-mediated, sugar-driven disease resulting in demineralisation and destruction of dental hard tissues, leading to cavitation if left untreated.
Replacement of Previous Prosthesis: The removal and substitution of an existing dental prosthesis due to wear, failure, poor fit, biological complications, aesthetic concerns, or changes in oral conditions.
Congenital Absence: The developmental failure of a tooth germ resulting in the absence of one or more teeth (hypodontia, oligodontia, or anodontia), present from birth and not due to extraction or disease.
Restoration cost: The restoration cost refers to the total monetary expenditure directly associated with the fabrication, delivery, and placement of a dental restoration intended to restore tooth form, function, or esthetics. This includes clinical procedures, laboratory fees, and materials required to complete the restorative intervention, as defined within the scope of *restoration* in the Glossary of Prosthodontic Terms.
Prosthetic cost: The prosthetic cost is the portion of treatment cost attributable specifically to the prosthesis itself, including its design, fabrication, laboratory processing, and delivery. In accordance with the GPT definition of *prosthesis* (“an artificial replacement for a missing part of the human body”), this cost excludes diagnostic, surgical, or preparatory procedures unless they are inseparable from the delivery of the prosthesis.
Total patient cost: The total patient cost represents the overall financial burden incurred by the patient for prosthodontic treatment. It encompasses all direct clinical, laboratory, prosthetic, and associated treatment costs required to achieve oral rehabilitation, consistent with comprehensive *prosthodontic treatment* as defined in the GPT.
Cost per restored tooth: The cost per restored tooth is a derived economic measure calculated by dividing the total cost of prosthodontic treatment by the number of teeth restored or replaced. This metric reflects the average financial investment per functional tooth restored and is used for comparative economic evaluation of prosthodontic rehabilitation outcomes.
Conventional Bridges (Fixed Partial Dentures): A conventional bridge, termed a *fixed partial denture* in the Glossary of Prosthodontic Terms, is a prosthesis that replaces one or more missing teeth and is cemented or otherwise rigidly retained on prepared natural teeth or abutments adjacent to the edentulous space.
Implants (Dental Implants): A dental implant is a prosthetic device made of biocompatible material that is surgically placed into the alveolar bone to provide support, retention, and stability for a dental prosthesis, as defined in the GPT.
Implants and Other Fixed Restorations: Implant-supported and other fixed restorations refer to non-removable prostheses that are supported by dental implants, natural teeth, or a combination thereof, and are rigidly attached through cementation or screw retention, restoring function and esthetics.
Implant-Supported Overdentures: An implant-supported overdenture is a removable prosthesis that replaces missing teeth and associated structures and is supported, retained, or stabilised by one or more dental implants, while also deriving support from the oral mucosa, consistent with the GPT definition of an overdenture.
Maryland Bridges (Resin-Bonded Fixed Partial Dentures): A Maryland bridge, classified in the GPT as a *resin-bonded fixed partial denture*, is a fixed prosthesis that replaces one or more missing teeth and is retained primarily by resin bonding to the enamel surfaces of adjacent teeth, typically with minimal tooth preparation.
Complete Dentures: A complete denture is a removable dental prosthesis that replaces all natural teeth and associated structures of the maxilla, mandible, or both, restoring function, esthetics, and phonetics, as defined in the Glossary of Prosthodontic Terms.
Partial/Telescopic Dentures: A removable partial denture is a removable prosthesis that replaces one or more, but not all, missing teeth in a dental arch and is supported by remaining teeth and/or soft tissues. A telescopic denture is a type of removable partial denture retained by a double-crown system consisting of primary copings on abutment teeth and secondary crowns within the prosthesis, providing frictional retention, consistent with GPT concepts of precision attachments and removable prostheses.
Removable Thermoplastic Prostheses: Removable thermoplastic prostheses are removable dental prostheses fabricated from flexible thermoplastic resin materials and function as removable partial dentures. While the material itself is not explicitly defined in the GPT, these prostheses fall within the GPT definition of removable partial dentures based on their function, support, and mode of retention.
