# Supplementary material for: Preliminary cost analysis of prosthodontic rehabilitation in partial and complete edentulism: evidence from a private clinical setting in Greece
Source: Front Public Health. 2026 Feb 4;14:1779013. doi: 10.3389/fpubh.2026.1779013 (PMC12913511; doi:10.3389/fpubh.2026.1779013)
Supplement: Supplementary file 1 [file Data_Sheet_1.docx]

**Supplementary Material**

**Preliminary cost analysis of prosthodontic rehabilitation in partial and complete edentulism: evidence from a private clinical setting in Greece**

**Supplementary Table A. Cost of dental restorations**

**Supplementary Table B. Dental laboratory costs**

**Supplementary Table C. Number of missing teeth versus number of teeth restored in the study population (N=76)**

**Supplementary Table A.** Cost of dental restorations

| Study | Country | Type of Work | Cost | Currency |
| --- | --- | --- | --- | --- |
| Pappa (2017) | Greece | Conventional bridge | 1,896.79 | € |
| Pappa (2017) | Greece | Implant-supported prosthesis | 1,826.19 | € |
| Brägger et al. (2005) | Switzerland | 3-unit fixed partial denture (FPD) | 3,939.40 | SFr |
| Brägger et al. (2005) | Switzerland | Single implant crown | 3,218.00 | SFr |
| Zitzmann et al. (2013) | Switzerland | Fixed bridge (FDP) | 5,082.00 | CHF |
| Zitzmann et al. (2013) | Switzerland | Implant crown (ISC) | 4,498.00 | CHF |
| Losenická et al. (2021) | Czechia | Fixed bridge | 40,161.00 | CZK |
| Losenická et al. (2021) | Czechia | Implant crown | 40,286.00 | CZK |
| Chun et al. (2016) | South Korea | Implant (direct cost) | 1,339.170 | KRW |
| Chun et al. (2016) | South Korea | Bridge (CFDP, direct cost) | 1,292.960 | KRW |
| Zhurakivska et al. (2023) | Italy | Complete denture | 1,804.40 | € |
| Zhurakivska et al. (2023) | Italy | Overdenture 2 implants | 4,473.20 | € |
| Zhurakivska et al. (2023) | Italy | Overdenture bar 2 implants | 4,608.80 | € |
| Zhurakivska et al. (2023) | Italy | Mini implants overdenture 4 implants | 4,742.00 | € |
| Zhurakivska et al. (2023) | Italy | Fixed denture 4 implants | 10,008.80 | € |
| Roy et al. (2020) | Netherlands | Complete denture | 1,058.00 | $ |
| Roy et al. (2020) | Netherlands | Overdenture 2 implants | 3.441,00 | $ |
| Roy et al. (2020) | Canada | Overdenture 1 implant | 957.14 | CAD |
| Roy et al. (2020) | Canada | Overdenture 2 implants | 1,678.64 | CAD |
| Roy et al. (2020) | UK | Overdenture 2 mini implants | 296.00 | £ |
| Roy et al. (2020) | UK | Overdenture 2 conventional implants | 688.00 | £ |
| Ghiasi et al. (2022) | Sweden | Overdenture 2 implants | 4,519.00 | € |
| Ghiasi et al. (2022) | Sweden | Fixed denture 4 implants | 9,341.00 | € |
| Ghiasi et al. (2022) | Sweden | Fixed denture 6 implants | 11,475.00 | € |
| Fueki et al. (2021) | Japan | Partial denture, thermoplastic | 1,222.00 | $ |
| Fueki et al. (2021) | Japan | Partial denture, metal clasp | 179.00 | $ |
| Anderson et al. (2020) | USA | Crown (metal) | 633.00 | $ |
| Anderson et al. (2020) | USA | Crown (metal-porcelain) | 633.00 | $ |
| Anderson et al. (2020) | USA | Crown (porcelain) | 642.00 | $ |
| Anderson et al. (2020) | USA | Veneers | 2,500.00 | $ |
| Anderson et al. (2020) | USA | Denture | 1,200.00 | $ |
| Anderson et al. (2020) | USA | Implant | 3,500.00 | $ |
| Korenori et al. (2018) | Japan | Implant | 2,744.00 | € |
| Korenori et al. (2018) | Japan | Bridge (insured) | 420.00 | € |
| Korenori et al. (2018) | Japan | Bridge (private) | 2,618.00 | € |
| Korenori et al. (2018) | Japan | Partial denture | 368.00 | € |

**Supplementary Table B.** Dental laboratory costs

| Study | Country | Type of Work | Cost | Currency |
| --- | --- | --- | --- | --- |
| Bessadet et al. (2025) | Switzerland | Single implant crown (hybrid) | 941.95 | CHF |
| Bessadet et al. (2025) | Switzerland | Single implant crown (conventional) | 1,245.65 | CHF |
| Bessadet et al. (2025) | Italy | Single implant restoration (digital) | 277.30 | € |
| Bessadet et al. (2025) | Italy | Single implant restoration (conventional) | 392.20 | € |
| Bessadet et al. (2025) | Switzerland | Single implant crown (digital) | 505.85 | CHF |
| Bessadet et al. (2025) | Switzerland | 3-unit implant bridge (digital, TRIOS 3) | 566.00 | CHF |
| Bessadet et al. (2025) | Switzerland | 3-unit implant bridge (digital, Virtuo Vivo) | 711.00 | CHF |
| Bessadet et al. (2025) | Switzerland | 3-unit implant bridge (hybrid) | 812.00 | CHF |
| Lo Russo et al. (2024) | Italy | Complete denture (conventional) | 270.00 | $ |
| Lo Russo et al. (2024) | Italy | Complete denture (digital milled) | 184.00 | $ |
| Lo Russo et al. (2024) | Italy | Complete denture (3D printed) | 67.00 | $ |

**Supplementary Table C.** Number of missing teeth versus number of teeth restored in the study population (N=76)

| **Missing teeth ↓** | **Teeth Restored →** | **Total patients ↓** |
| --- | --- | --- |
|  | 1 2 3 4 5 6 7 8 9 1 0 1 1 1 2 1 4 1 7 2 0 28 |  |
| 1 | 10 1 8 1 0 0 0 0 0 1 0 0 0 0 0 0 | 21 |
| 2 | 0 6 1 3 1 0 0 1 0 0 1 0 0 0 0 0 | 13 |
| 3 | 0 0 3 0 1 0 0 0 0 0 0 0 0 0 0 0 | 4 |
| 4 | 0 0 1 1 0 0 0 0 0 0 0 1 0 0 0 0 | 3 |
| 5 | 0 0 0 1 0 0 0 0 0 0 0 0 0 0 0 1 | 1 |
| 6 | 0 0 0 0 0 1 0 0 0 0 0 0 0 0 0 1 | 1 |
| 7 | 0 0 0 0 0 0 1 0 0 0 0 0 0 0 0 2 | 2 |
| 8 | 0 0 0 0 0 0 1 0 0 0 0 0 0 0 0 1 | 1 |
| 10 | 0 0 0 0 0 0 0 3 0 1 0 0 0 0 0 0 | 4 |
| 11 | 0 0 0 0 0 0 0 1 0 0 0 0 0 0 0 0 | 1 |
| 12 | 0 0 0 0 0 0 0 0 0 1 0 0 0 0 0 0 | 1 |
| 14 | 0 0 0 0 0 0 0 0 0 1 0 1 0 0 0 0 | 2 |
| 15 | 0 0 0 0 0 0 0 0 1 0 0 0 0 0 0 0 | 1 |
| 16 | 0 0 0 0 0 0 0 0 0 0 0 0 1 1 0 0 | 2 |
| 18 | 0 0 0 0 0 0 0 0 0 0 0 0 1 2 0 0 | 3 |
| 19 | 0 0 0 0 0 0 0 0 0 1 0 0 1 0 0 0 | 2 |
| 20 | 0 0 0 0 0 0 0 0 0 0 0 0 2 0 0 0 | 2 |
| 28 | 0 0 0 0 0 0 0 0 0 0 0 0 0 0 0 1 | 1 |
| 32 | 0 0 0 0 0 0 0 0 0 0 0 0 0 0 0 2 | 4 |
| **Total** | **10 7 13 6 2 1 2 5 1 5 1 3 8 1 1 3** | **76** |
